# Serum 25-hydroxyvitamin D concentrations and postmenopausal breast cancer risk: a nested case control study in the Cancer Prevention Study-II Nutrition Cohort

**DOI:** 10.1186/bcr2356

**Published:** 2009-08-28

**Authors:** Marjorie L McCullough, Victoria L Stevens, Roshni Patel, Eric J Jacobs, Elizabeth B Bain, Ronald L Horst, Susan M Gapstur, Michael J Thun, Eugenia E Calle

**Affiliations:** 1 Epidemiology Research, American Cancer Society, 240 Williams Street, Atlanta, GA 30303-1002, USA; 2 Heartlands Assays, Inc. 2325 N. Loop Drive, Suite 6300, Ames, IA 50010, USA

## Abstract

**Introduction:**

Vitamin D status measured during adulthood has been inversely associated with breast cancer risk in some, but not all, studies. Vitamin D has been hypothesized to prevent breast cancer through genomic and non-genomic actions in cell-cycle regulation.

**Methods:**

A subset (n = 21,965) of female participants from the prospective Cancer Prevention Study-II (CPS-II) Nutrition Cohort provided a blood sample from 1998-2001 and were followed through 2005. We measured serum 25-hydroxyvitamin D (25(OH)D) in 516 verified incident cases and 516 controls, matched on birth date (± 6 months), date of blood draw (± 6 months) and race. Information on medical history, risk factors and lifestyle was available from repeated questionnaires. We computed multi-variable odds ratios (OR) and 95% confidence intervals (95% CI) for the association between 25(OH)D quintile and breast cancer risk using unconditional logistic regression, controlling for matching factors and additional confounders.

**Results:**

We observed no association between 25(OH)D and breast cancer (OR = 1.09, 95% CI 0.70-1.68, *P *= 0.60) for the top vs bottom quintile. Using *a priori *cut-points, the OR was 0.86 (95% CI 0.59-1.26), for ≥75 vs <50 nmol/L. Results were not different when the first two years of follow-up were excluded, or in analyses stratified by season, latitude, BMI, postmenopausal hormone use, or by tumor grade or estrogen receptor status.

**Conclusions:**

These results do not support an association between adulthood serum 25(OH)D and postmenopausal breast cancer. We cannot rule out an association with 25(OH)D status earlier in life.

## Introduction

Although breast cancer death rates have declined in recent years, breast cancer remains the most commonly diagnosed cancer and the second leading cause of cancer death in women in the US. [1]. Risk factors include family history, high breast tissue density, long menstrual history (menstrual periods that begin early and end late in life), nulliparity, late age at first birth, use of hormone replacement therapy, regular alcohol consumption, low physical activity, high body mass index (BMI) and weight gain during adulthood (for postmenopausal breast cancer). Apart from energy imbalance, dietary risk factors have not been consistently associated with risk of the disease [2].

Breast cancer rates are higher in areas of the US with lower overall ultraviolet B radiation exposure, raising the possibility that higher levels of vitamin D could reduce breast cancer risk [3]. Vitamin D may regulate cell proliferation and differentiation through both genomic and non-genomic effects [4]. Epidemiologic studies have observed lower breast cancer risk with greater self-reported [5] and measured [6] sun exposure. The association between dietary and supplemental intake of vitamin D with breast cancer risk has been inconsistent [7], potentially because typical doses of vitamin D intake have comparatively less impact on vitamin D status than ultraviolet B exposure, and because of measurement error in nutrition assessment. Circulating 25-hydroxyvitamin D (25(OH)D) reflects an input of vitamin D from both sun exposure and diet over the preceding several weeks, and thus represents an integrated measure of vitamin D status. This metabolite is thought to be relevant because it provides substrate for tissue-specific synthesis of the active form of vitamin D (1,25-dihydroxyvitamin D (1,25(OH)_2_D)), the natural ligand for the nuclear vitamin D receptor. Although three case-control studies reported an inverse relation between circulating 25(OH)D and breast cancer risk [8-10], results from three nested case-control studies where blood was collected prior to diagnosis were less consistent [11-13].

If vitamin D status is associated with a lower risk of breast cancer, this would represent an easily modifiable risk factor. To contribute to the limited and inconsistent prospective data on this topic of important public health interest, we examined circulating 25(OH)D concentrations in a nested case-control study of over 500 postmenopausal breast cancer cases and their matched controls from the Cancer Prevention Study-II (CPS-II).

## Materials and methods

### Study population

Women in this analysis were participants in the CPS-II Nutrition Cohort, a prospective study of cancer incidence among men and women from 21 states in the US, established by the American Cancer Society in 1992 [14]. At enrollment, 86,404 men and 97,788 women completed a 10-page, self-administered questionnaire on medical, dietary, reproductive, and lifestyle variables. Beginning in 1997, follow-up questionnaires were sent to cohort members every two years to update exposure information and to ascertain newly diagnosed cancers. All aspects of the CPS-II cohort were approved by the Emory University (Atlanta, GA, USA) Institutional Review Board.

### Breast cancer nested case-control study

From June 1998 to June 2001, blood specimens were collected from 21,965 women in the Nutrition Cohort who lived near a major collaborating hospital or medical clinic in their community, as described in detail elsewhere [14]. All participants completed a brief questionnaire and provided informed consent at the time of blood draw. We collected a maximum of 43 mL of nonfasting whole blood from each participant drawn in two 15 mL tubes containing the anticoagulant ethylenediamine tetraacetic acid and a 13 mL serum separator tube. Hospital staff centrifuged the serum separator tubes to separate serum from cellular blood components prior to shipping, and all samples were then shipped in foam containers with coolant packs overnight by Express Mail to a central repository in Rockville, Maryland. Shipments of blood from the field were received, unpacked, aliquoted, and frozen in liquid nitrogen vapor phase at approximately -130°C for long-term storage [14].

Eligible cases included women who reported a new diagnosis of breast cancer on a biennial CPS-II Nutrition Cohort Survey between the date of their blood draw and 30 June, 2005 (n = 514) or who did not report an incident breast cancer but for whom fatal breast cancer was identified through linkage with the National Death Index [16] (n = 2). We asked women reporting a diagnosis for permission to review their medical records to confirm their diagnosis. If medical records were not available, we contacted state cancer registries. Previous pilot work in this cohort found that the sensitivity of self-reported breast cancer was 91% [15] suggesting we successfully ascertained incident breast cancer cases in the cohort.

We initially identified a total of 535 breast cancers diagnosed after the date of blood draw. We excluded women who were pre- or peri-menopausal (n = 3) at baseline, those with no appropriate match (n = 1), women with one or less vial of serum (n = 14), and one woman with an extreme 25(OH)D level (more than three standard deviations above the mean). The analysis is based on 516 cases and on an equal number of matched controls, aged 47 to 85 years at baseline. The duration of follow-up between the date of blood draw and breast cancer diagnosis ranged from less than 1 month to 6.9 years.

We documented the estrogen receptor (ER) and progesterone receptor (PR) status of the tumors from medical record reports and through state cancer registries, when available. Information on ER and PR status was available for 76% and 73% of cases, respectively.

### Control selection

Each control was individually matched to a case on birth date (± 6 months), race/ethnicity (Caucasian, African-American, other/unknown), and date of blood collection (± 6 months). We randomly selected one postmenopausal female control who had provided a blood sample and was cancer free (except for non-melanoma skin cancer) at the date of the diagnosis of the case. For two cases of 'other' race, an appropriately matched control was not available, and they were matched to a Caucasian control.

### Vitamin D assays

Laboratory analyses were conducted at Heartland Assays, Inc (Ames, IA, USA). Case-control pairs were analyzed in the same batch in no particular order. A direct, competitive chemiluminescence immunoassay using the DiaSorin LIAISON platform, as described in detail elsewhere [17], was used to quantitate 25(OH)D levels.

### Quality control

An average of five quality control samples were included in each batch of 100 samples. These quality control samples were from four separate sources: a pooled sample of non-multivitamin users, a pooled sample of multivitamin users, and two individual CPS-II Nutrition Cohort participants.

Coefficients of variation (CV) for duplicate serum aliquots included in all laboratory sample batches were calculated for each of the four quality control samples, and ranged from 6.4 to 8.8%. Intra-batch and inter-batch CVs were 5.4% and 8.1%, respectively.

### Measurement of covariates

Information on breast cancer risk factors and potential confounders was ascertained from responses to biennial questionnaires from the CPS-II cohort. For the current analysis, we obtained information on current BMI, date of blood draw, and use of individual vitamin D supplements from the brief questionnaire completed at blood draw. Information on reproductive risk factors, history of benign breast disease, family history and education was assessed on earlier questionnaires. Other variables, including alcohol use, postmenopausal hormone use, diet, recreational physical activity and zip code (for latitude) were collected from the comprehensive 1999 questionnaire. For these variables, 24 cases (5%) completed the questionnaire after diagnosis of breast cancer. Sensitivity analyses removing these cases and their controls did not change the risk estimates.

### Data analysis

Circulating 25(OH)D (nmol/L) was normally distributed and was not improved with log transformation; thus, we used untransformed values. We divided participants into quintiles based on the distribution of serum 25(OH)D in the control group. We observed the expected differences in 25(OH)D concentrations by season of blood draw: mean levels (± standard deviation) were 49.1 (± 21.2) in Winter (December to February), 53.7 (± 25.1) in Spring (March to May), 58.1 (± 22.2) in Summer (June to August) and 59.5 (± 20.3) in Fall (September to November). For stratified analyses, we classified season according to two general seasons: a 'warm' season (June to November) and a 'cool' season (December to May). Independent correlates of 25(OH)D among controls in our study were evaluated using linear regression models.

Our primary statistical approach used unconditional logistic regression to estimate the odds ratio (OR) and 95% confidence intervals (CI) for risk of cancer comparing quintiles 2 to 5 with the lowest quintile, to preserve statistical power when conducting stratified analyses on factors that were not matched by design. In sensitivity analyses, we also conducted conditional logistic regression on main effects models and results were similar. We first assessed the relation between serum 25(OH)D and breast cancer risk adjusting only for matching factors (date of birth ± six months, date of serum collection ± six months, race (white, other)).

In addition to controlling for matching factors, we included as covariates those factors that were correlated with serum 25(OH)D concentrations and/or related to breast cancer risk in this sample. These included: season of serum collection (Winter/Spring/Summer/Fall); weight change since age 18 years (lost >5 kg, lost or gained <5 kg, gained 5 to <10, 10 to <20, 20 to <30, and 30+ kg, and missing data); body mass index (<22.5, 22.5 to <25.0, 25.0 to <30 (and missing data), 30.0+), and combinations of number of live births (nulliparous, 1 to 2, 3+) and age of mother at first birth (<25 or ≥25). Adjustment for alcohol consumption (non-drinker, <1 drink/day, 1+ drink/day, missing data); recreational physical activity in metabolic equivalents (MET) hours per week (quartiles); family history of breast cancer (yes, no); history of benign breast disease (yes, no); age at menarche (<12, 12, 13 (and missing data), >13 years); age at menopause (<45, 45-<50, 50-<54, 54+ years), and postmenopausal hormone use (never, former, current) did not change the effect estimates and therefore were not included. To test for trend, we created a continuous variable consisting of the median 25(OH)D value from each quintile, and included this variable in the regression model.

We additionally examined the association between 25(OH)D and breast cancer risk using *a priori *cut-points of less than 50 nmol/L as deficiency (referent), 50 to less than 75 nmol/L as 'insufficient', and 75+ nmol/L as 'adequate' [18]. We examined the possibility of a non-linear relation between circulating 25(OH)D and breast cancer risk using restricted cubic splines [19]. The linearity of the association between 25(OH)D and breast cancer risk was tested using the likelihood ratio test, comparing the model with only the linear term to the model with the linear and the cubic spline terms.

We conducted stratified analyses comparing breast cancer risk across tertiles of 25(OH)D concentration within strata of postmenopausal hormone use (never and ever use), BMI (<25 and ≥25 kg/m^2^), time of year of blood draw ('warm' and 'cool' seasons), latitude (above and ≤37°), tertile of total calcium intake, multivitamin supplement use (assessed in 1999) and use of individual vitamin D supplements (yes, no) reported at blood draw. Interaction terms were cross-product terms of the tertile measure (0, 1, 2, in continuous form) and dummy variables for potential effect modifiers. Multiplicative interactions were evaluated using the likelihood ratio test.

We conducted sensitivity analyses excluding cases diagnosed during the first two years of follow-up, and using season-specific 25(OH)D quintile cut-points [20]. We also evaluated the association between 25(OH)D and breast cancer according to ER status of the tumor, and according to tumor invasiveness (*in situ*, local, regional/distant). All statistical analyses were conducted using SASv.9.2 (SAS Institute, Inc., Cary, NC, USA).

## Results

Characteristics of the postmenopausal breast cancer cases and controls are provided in Table 1. The age and race of participants were similar, as expected based on matching criteria. Although the mean BMI was not different between cases and controls, breast cancer cases had gained more weight since age 18 compared with controls. A greater percentage of cases reported a family history of breast cancer, a personal history of benign breast disease, and a later age at menopause. Controls had more children earlier in life compared with cases. There was no difference in the percentage of cases and controls currently or formerly taking postmenopausal hormones. A slightly higher (non-significant) percentage of cases consumed more than one alcoholic drink per day. Recreational physical activity and use of nutritional supplements were not appreciably different between cases and controls.

**Table 1:** Baseline characteristics in cases and controls, CPS II Nutrition Cohort Women (1999 to 2005)*

**Characteristic**	**Cases (n = 516)**	**Controls (n = 516)**	***P *value^‡‡^**
	Mean (SD)		
Age at serum collection (year)	69.5 (5.9)	69.6 (5.8)	0.7
Plasma 25(OH)D (nmol/L)	56.5 (22.0)	56.2 (22.2)	0.8
Latitude (degree)	39.0 (4.7)	39.3 (4.8)	0.2
BMI at blood collection (kg/m^2^)	25.9 (4.9)	25.8 (4.8)	0.9
Weight gain since age 18 (kg)	14.1 (15.5)	11.1 (18.3)	0.006
Exercise METS^†^	15.4 (13.8)	15.6 (14.7)	0.9
Age at menarche (years)	12.7 (1.5)	12.7 (1.4)	0.6
Age at menopause (years)	48.6 (6.1)	47.8 (6.0)	0.03

	Percentage (%)		
Family history of breast cancer	23.6	16.9	0.007
History of benign breast disease	33.9	27.5	0.01
Race			
White	96.7	97.1	0.7
Other (Black/Hispanic/Oriental)	3.3	2.9	
College graduate	24.2	23.4	0.5
Parity and age at first birth			
Nulliparous	9.1	8.5	0.006
<25, 1 to 2 live births	13.2	12.4	
25+, 1 to 2 live births	22.5	14.7	
<25, 3+ live births	35.3	45.0	
25+, 3+ live births	20.0	19.4	
Postmenopausal hormone use			
Never	22.1	25.4	0.4
Former	58.1	53.1	
Current	16.1	16.7	
Season of blood collection			
Dec to Feb	15.1	18.2	0.1
Mar to May	18.8	17.4	
Jun to Aug	35.1	29.5	
Sep to Nov	31.0	34.9	
Alcohol intake (≥ 1 drink/day)	21.3	16.1	0.1
Multivitamin use containing vitamin D^‡^			
Not current user	30.8	34.9	0.6
Current user	63.6	59.6	
Calcium supplement use			
Not current user	32.4	32.9	0.5
Current user	59.9	57.3	
Vitamin D supplement use^§^			
Not current user	54.1	53.7	0.9
Current user	16.1	16.9	

	Mean (SD)		
**Dietary variables^#^**			
Total calcium (mg/day)	1358.0 (565.2)	1293.7 (576.6)	0.08
Dietary calcium (mg/day)	777.0 (285.3)	768.6 (283.0)	0.6
Supplemental calcium (mg/day)	581.0 (504.3)	515.0 9493.4)	0.08
Total vitamin D (IU/day)	439.5 (243.6)	415.0 (239.7)	0.1
Dietary vitamin D (IU/day)	192.0 (105.3)	187.6 (96.1)	0.5

* Standardized to age distribution of the study population.

^† ^Metabolic equivalent of task (METS) are defined for each type of exercise-related physical activity as a multiple of metabolic equivalent of sitting quietly for one hour.

^‡ ^Reported on the 1999 questionnaire.

^§ ^Reported at blood collection.

^# ^All dietary variables are adjusted for energy intake which was estimated from the 1992 brief Block Food Frequency Questionnaire (FFQ).

^‡‡†† ^*P *values for categorical variables are based on the chi-squared test and P values for continuous variables are based on the t-test for difference of means.

25(OH)D = 25-hydroxyvitamin D; BMI = body mass index; CPS-II = Cancer Prevention Study-II; SD = standard deviation.

Several factors predicted circulating 25(OH)D among controls (results not shown). Winter season predicted significantly lower 25(OH)D concentrations compared with Summer. Current BMI and weight gain each individually predicted 25(OH)D, but when considered simultaneously, current BMI was the stronger predictor. Recreational physical activity was associated with significantly higher concentrations, likely due to greater sunlight exposure during outdoor exercise. Total vitamin D intake (which was not considered as a confounder due to potential over-control) was a statistically significant predictor of 25(OH)D in univariate and multivariate models. An increment of 800 IU vitamin D corresponded to a 16 nmol/L higher circulating 25(OH)D in both models (not shown).

**Figure 1:**
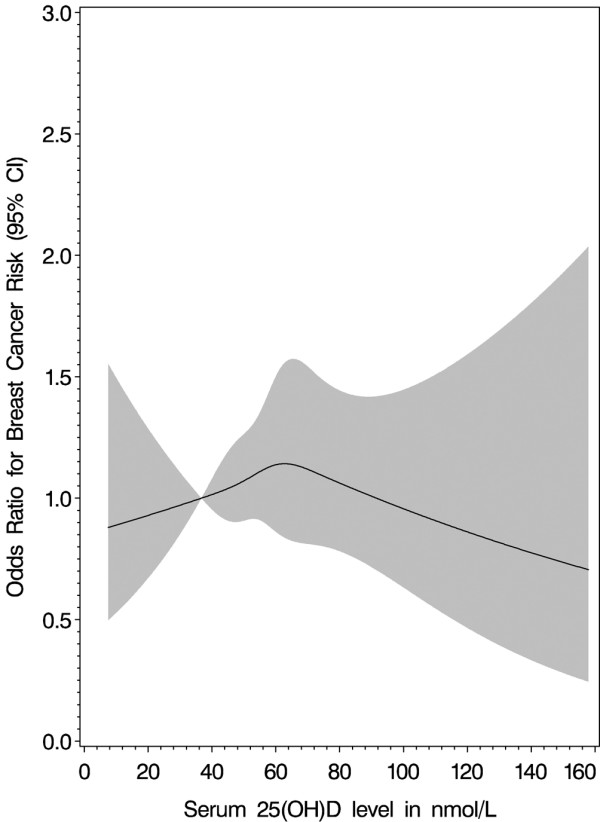
Restricted cubic spline showing the fully adjusted associations between serum 25(OH)D levels and breast cancer risk in 1032 participants in the Cancer Prevention Study-II Nutrition Cohort. The reference value is less than 36.7 nmol/L. The knots are defined by the quintile cut points for 25-hydroxyvitamin D (25(OH)D) and are 36.7, 49.8, 60.8, and 73.2.

Table 2 presents the OR and 95% CI for the association between quintile of 25(OH)D and postmenopausal breast cancer risk. Compared with women in the lowest 25(OH)D quintile (<36.7 nmol/L), the OR for women in the highest quintile (>73.2 nmol/L) was 1.09 (0.70 to 1.68; *P *= 0.6). A cubic spline graph (Figure 1) illustrates this association with knots forced at quintile cut-points. An apparent inverse U-shaped association was not statistically significantly non-linear (*P *= 0.54). Results were similar when 227 cases diagnosed in the first two years after blood draw were excluded (OR for fifth vs first quintile = 1.04, 95% CI 0.62 to 1.75, not shown). Results using season-specific 25(OH)D cut-points were also similar (OR for fifth vs first quintile = 1.13, 95^th ^CI 0.74-1.72). In sub-analyses using absolute cut-points of 75 or more vs less than 50 nmol/L, the ORs remained null for all cases (0.86, 95% CI 0.59 to 1.26; *P *= 0.7).

**Table 2:** Odds ratio (OR) and 95% confidence interval (CI) between 25(OH)D levels and breast cancer risk, CPS II Nutrition Cohort women

	**Quintiles of serum 25(OH)D concentration (nmol/L)**					***P *for trend^‡^**
	**<36.7**	**36.7-<49.8**	**49.8-<60.8**	**60.8-<73.2**	**73.2+**	
All cases (n)	89	115	99	118	95	
Controls (n)	104	102	105	102	103	
OR (95% CI)*	1.00 (-)	1.31 (0.88-1.94)	1.11 (0.74-1.66)	1.35 (0.91-2.01)	1.07 (0.71-1.61)	0.8
OR (95%CI)^†^	1.00 (-)	1.29 (0.86-1.94)	1.14 (0.75-1.72)	1.44 (0.96-2.18)	1.09 (0.70-1.68)	0.6

	**Tertiles of serum 25(OH)D concentration (nmol/L)**			***P *for trend^‡^**		
	**<45.9**	**45.9-<64.2**	**64.2+**			
**ER+ cases (n)**	102	120	120			
**Controls (n)**	177	160	179			
OR (95% CI)*	1.00 (-)	1.32 (0.94-1.87)	1.16 (0.82-1.63)	0.4		
**OR (95%CI)^†^**	1.00 (-)	1.32 (0.93-1.88)	1.15 (0.80-1.65)	0.5		
**ER- cases (n)**	17	16	16			
**Controls (n)**	177	160	179			
OR (95% CI)*	1.00 (-)	1.02 (0.49-2.12)	0.90 (0.44-1.87)	0.8		
**OR (95%CI)^†^**	1.00 (-)	1.03 (0.48-2.19)	0.95 (0.43-2.06)	0.9		
***In situ *cases (n)^§^**	34	39	30			
**Controls (n)**	177	160	179			
OR (95% CI)*	1.00 (-)	1.29 (0.77-2.16)	0.89 (0.52-1.54)	0.7		
**OR (95%CI)^†^**	1.00 (-)	1.23 (0.72-2.09)	0.87 (0.49-1.55)	0.7		
**Localized cases (n)^§^**	90	114	114			
**Controls (n)**	177	160	179			
OR (95% CI)*	1.00 (-)	1.44 (1.01-2.06)	1.25 (0.88-1.79)	0.2		
**OR (95%CI)^†^**	1.00 (-)	1.45 (1.01-2.09)	1.30 (0.90-1.89)	0.2		
**Regional/Distant Cases (n)^§^**	35	25	28			
**Controls (n)**	177	160	179			
OR (95% CI)*	1.00 (-)	0.79 (0.45-1.38)	0.79 (0.46-1.37)	0.4		
**OR (95%CI)^†^**	1.00 (-)	0.78 (0.43-1.41)	0.75 (0.41-1.37)	0.3		

* Unconditional logistic regression adjusted for birth year, year of blood draw, race, and season.

^† ^Unconditional logistic regression adjusted for birth year, year of blood draw, race, season, parity and age at first birth, body mass index at blood collection and weight change from age 18 years to blood collection.

^‡ ^*P *value for trend is calculated using the median for each category and modeled as a continuous variable.

^§ ^Definition of cases based on Surveillance Epidemiology and End Results (SEER) summary staging.

25(OH)D = 25-hydroxyvitamin D; CPS-II = Cancer Prevention Study-II; CI = confidence interval; ER = estrogen receptor; OR = odds ratio.

Associations by ER status and tumor subtype were examined using tertiles of 25(OH)D concentration to allow comparison among groups with smaller numbers (Table 2). There were no differences by ER status of the tumor, and addition of PR status combined with ER status did not meaningfully affect the results. Likewise, there were no differences in results when tumors were classified as *in situ*, localized, or regional/distant. In sub-analyses using absolute cut-points of 75 or more vs less than 50 nmol/L; ORs (± 95 CI) were: ER+, OR = 0.86, 95% CI 0.56 to 1.32, *P *= 0.8; ER-, OR = 0.90, 95% CI 0.35 to 2.30, *P *= 1.0; *in situ*, OR = 0.64, 95% CI 0.32 to 1.30, *P *= 0.3; localized, OR = 1.16, 95% CI 0.76 to 1.78, *P *= 0.3; regional and distant, OR = 0.39, 95% CI 0.16 to 0.92, *P *= 0.08. Thus, when using established cut-points, individuals who had sufficient vs. deficient levels were at borderline lower risk for regional/distant tumors. When modeled by each 10 nmol/L change in 25(OH)D, the multivariate-adjusted ORs and 95% CIs were as follows: all cases (1.01, 0.95 to 1.07); ER+ cases (1.02, 0.96 to 1.09); ER- cases (0.91, 0.78 to 1.06); *in situ *cases (0.99, 0.90 to 1.09); localized cases (1.04, 0.97 to 1.10) and regional/distant cases (0.93, 0.83 to 1.05).

The association between 25(OH)D and breast cancer risk did not vary by history of postmenopausal hormonal therapy, BMI, weight gain, 'warm' and 'cool' season of blood draw, or calcium intake. A significant interaction was observed by latitude: the OR among women living at 37° or more latitude who were in the top tertile of 25(OH)D (64 nmol/L+) vs bottom tertile (<46 nmol/L) was 0.35 (95% CI 0.17 to 0.73), vs OR = 1.07 (95% CI 0.59 to 1.95) among women living at <37° (*P *interaction = 0.003). The relation did not vary by use of vitamin D-containing multivitamin supplements (measured in 1999), but a significant interaction was observed for use of individual vitamin D supplements (yes, no) at blood draw: among women who reported not using vitamin D supplements, the OR for the top vs bottom tertile was 0.82 (95% CI 0.53 to 1.27), whereas among current users it was 1.82 (0.61 to 5.39; *P *interaction = 0.047).

## Discussion

In this nested case-control study of mostly Caucasian women, serum 25(OH)D concentrations assessed up to six years prior to diagnosis were not associated with risk of postmenopausal breast cancer. This finding did not change when we excluded cases occurring in the first two years after blood draw, and were not significantly different in analyses stratified by BMI, postmenopausal hormone use, season of blood draw, calcium intake, or multivitamin use. Likewise, 25(OH)D concentrations were not significantly associated with cancer subgroups defined on the basis of ER/PR status, or tumor stage/grade.

Our findings contribute to the relatively few prospective studies published on this topic [11-13]. In a nested case-control analysis of 701 cases and 724 controls from the Nurses' Health Study, Bertone and colleagues [11] observed a borderline 27% lower relative risk of breast cancer comparing women in the top vs bottom 25(OH)D quintile (*P *= 0.06). In contrast, among 1005 postmenopausal cases and 1005 controls from the Prostate, Lung, Colorectal and Ovarian Cancer Screening Trial [13], no association between prediagnostic 25(OH)D and breast cancer risk was found (OR = 1.04, 95% CI 0.75 to 1.45), comparing top (≥84 nmol/L) to bottom (<45.8 nmol/L) quintile. A prospective analysis from the NHANES study [12] reported a relative risk for fatal breast cancer (n = 28 cases) among women with 62.5 nmol/L or higher vs less than 62.5 nmol/L circulating 25(OH)D of 0.28 (95% CI 0.08 to 0.93; *P *trend of continuous 25(OH)D = 0.76) [12]. All three published case-control studies [8-10] reported statistically significant inverse associations between circulating 25(OH)D and breast cancer risk. However, because blood was drawn after cancer diagnosis, it is possible that cancer diagnosis may have influenced circulating 25(OH)D through treatment or behavioral changes. Finally, reports from two randomized controlled trials of vitamin D and/or calcium supplementation were indeterminate [21,22]. A seven-year randomized trial observed no difference in breast cancer risk for women receiving 400 IU vs placebo [21], but the intervention study dose was relatively low and a high percentage of women in the control group began taking supplements during the trial. The other study found a lower risk of overall cancer with the use of 1100 IU vitamin D in a secondary analysis from an osteoporosis trial [22], but there were too few breast cancers to examine this outcome separately.

Our study did not indicate the existence of a threshold for a protective association with breast cancer with circulating 25(OH)D, which has been hypothesized by some [23]. In the Nurses' Health Study, risk was lower starting at an approximate threshold of 70 to 80 nmol/L (different cut-points were employed by batch) [11]. Similar to our findings, Freedman and colleagues [13] found no association overall, with a non-significant slightly higher breast cancer risk in the middle quantiles of circulating 25(OH)D. Because the shape of the 25(OH)D - breast cancer association in the current study indicated a lower risk at both ends of the distribution, ORs were sensitive to the cut-points used. For example, in analyses using quintiles, 25(OH)D concentrations in the middle quantiles were associated with slightly higher risk. However, when we used *a priori *cut-points for deficiency, insufficiency and sufficiency [18], which resulted in shifting the referent group to higher 25(OH)D concentrations, there was no suggestion of increased risk in any category.

Body fat and weight gain were moderate negative confounders in our study (with the net effect of raising the OR when added to either the age-adjusted model or multivariate models), and BMI independently predicted circulating 25(OH)D. Body fat is a large storage reservoir for 25-OH-vitamin D, and lower circulating 25(OH)D in obese individuals is thought to be related to greater uptake into fat tissues, rather than lower sun exposure or less effective vitamin D synthesis [24]. In the Women's Health Initiative [21], baseline 25(OH)D was associated with a significantly lower risk of breast cancer, until models were further adjusted for BMI and physical activity. Studies of vitamin D and cancer risk need to carefully examine and control for potential confounding by obesity.

We found that 25(OH)D-breast cancer associations were modified by individual vitamin D supplement use at blood draw, with an inverse association among non-current vitamin D supplement users and a positive association among current supplement users (*P *interaction = 0.047). Reasons for this apparent interaction are unclear. It is possible that the source of vitamin D may be important. For example, at any given level of serum 25(OH)D, non-supplement users obtain a higher proportion of their vitamin D from cutaneous synthesis, and/or greater intake of fortified milk or fish than do supplement users. Vitamin D in the diet is strongly correlated with dietary calcium, which also may play a role in preventing breast carcinogenesis [25,26]. We also observed a significant inverse association between 25(OH)D and breast cancer risk among women living in northern (>37°), but not southern (≤37°) latitudes. Over 95% of women in this analysis living in the north were born in the north, whereas 64% of the women living at 37° or lower latitude were born in the south. It is possible that low 25(OH)D concentrations among women living in the north correlate better with chronically low concentrations. However, both of these interactions may also be due to chance.

A potential explanation for our null findings is that we missed the etiologically relevant time frame of vitamin D exposure for breast carcinogenesis. Several studies observed stronger inverse associations between greater intake of vitamin D and/or calcium in relation to mammographic density or breast cancer among premenopausal women [9,25,27-29] than postmenopausal women. In addition, self-reported sun exposure earlier in life was more strongly associated with postmenopausal breast cancer than sun exposure later in life [30]. We were unable to examine the relation between 25(OH)D and premenopausal breast cancer, or earlier life vitamin D exposure in relation to postmenopausal breast cancer. Additional limitations include a lower range of circulating 25(OH)D compared with some other studies [8,11,13], limiting our ability to examine higher cut-points of 25(OH)D status. This study also had a relatively small sample size, particularly when examining associations by tumor receptor status and subtype.

An advantage of this study is the use of an objective biomarker of vitamin D status, which reflects the integration of dietary and supplemental vitamin D intake as well as cutaneous synthesis. It is possible, however, that our single measure of 25(OH)D did not correctly classify women according to their usual, year-round vitamin D status. Circulating 25(OH)D reflects exposure to sun and diet from approximately the prior two months. Further, 25(OH)D fluctuates according to season of the year. To minimize this extraneous source of variation, we controlled for season of blood draw, and in sensitivity analyses, we used season-specific cut-points. Analyses stratified by season of blood draw were not statistically different.

## Conclusions

Our findings do not support an association between adulthood 25(OH)D status and postmenopausal breast cancer risk. Future studies with the ability to examine a wider distribution of 25(OH)D, vitamin D status earlier in life, and with greater statistical power to examine tumor subtypes and stage would add importantly to the literature.

## Abbreviations

1,25(OH)_2_D: 1,25-dihydroxyvitamin D; 25(OH)D: 25-hydroxyvitamin D; BMI: body mass index; CPS-II: Cancer Prevention Study-II; CI: confidence interval; CV: coefficient of variation; ER: estrogen receptor; METS: Metabolic equivalent of task; OR: odds ratio; PR: progesterone receptor; SD: standard deviation.

## Competing interests

The authors declare that they have no competing interests.

## Authors' contributions

MLM conceived of the study, participated in the design, and drafted the manuscript. VLS participated in the design, coordinated laboratory sample processing, and contributed to drafting the manuscript. RP participated in study design and conducted statistical analyses. EBB participated in study design and processed laboratory samples. EJJ participated in the design and contributed to drafting the manuscript. RLH conducted laboratory analyses and contributed to sections of manuscript draft. SMG participated in the design and contributed to drafting the manuscript. MJT contributed to drafting the manuscript. EEC participated in the design of the study. All authors read and approved the final manuscript.
